# A Memory-Efficient Transmission Scheme for Multi-Homed Internet-of-Things (IoT) Devices

**DOI:** 10.3390/s20051436

**Published:** 2020-03-06

**Authors:** Jaehyun Hwang, Joon Yoo

**Affiliations:** 1 Department of Computer Science, Cornell University, 430 Gates Hall, Ithaca, NY 14853, USA; jaehyun.hwang@cornell.edu; 2 Department of Software, Gachon University, 1342 Seongnamdaero, Seongnam 13120, Korea

**Keywords:** Multipath TCP, IoT, low-memory, packet scheduling

## Abstract

Internet-of-things (IoT) is a wide spreading technique that enables intelligence to the everyday objects, however, IoT devices are limited in computation and memory space due to their small physical sizes. As a result, IoT applications generally connect to the remote cloud that provides high computation and large storage. To enhance this communication, some IoT devices are equipped with multiple networks, e.g., cellular or Wi-Fi, by using Multipath TCP (MPTCP). However, MPTCP requires large buffer memory space compared to the legacy TCP, which is problematic for low-memory IoT devices. This paper proposes a new MPTCP scheme that leverages the multi-homed feature of low-memory IoT devices. Our design utilizes an application-level distributor that transmits packets to each MPTCP socket at each endpoint of the data. This scheme cleverly avoids the buffer blocking problem, while still maintaining the benefits of multi-homing. The main contribution of our paper is three-fold. First, our proposal achieves the benefits of multipath while avoiding buffer blocking due to out-of-order packets. Second, since our scheme utilizes the original MPTCP and modifies only the application level, it can be deployed more easily to the legacy systems. Finally, our experimental results, conducted on a Linux testbed and real-world cellular/Wi-Fi, show that the proposed scheme requires only half or less memory to achieve the performance of MPTCP.

## 1. Introduction

The recent proliferation of the Internet-of-Things (IoT) systems [1] has enabled intelligence to our everyday lives by leveraging inexpensive IoT devices. Since these small IoT devices are limited in computation and memory space, they typically communicate with the powerful backend servers and cloud for processing and storage [2]. Some IoT devices, such as home gateways, smart phones, smart watches, and connected cars, are equipped with multiple network interfaces, such as Digital Subscriber Line (DSL), cellular (e.g., NB-IoT, LTE-M, 3G, private LTE, etc.), and Wi-Fi, Bluetooth [3,4], thus enabling multi-homed Internet connectivity. Multipath TCP (MPTCP) [5] is the *de-facto* multipath standard, which was proposed as an extension to the TCP. MPTCP can exploit the multi-homing feature to provide benefits to the IoT devices such as higher throughput from multipath bandwidth, robust communication due to failure tolerance.

Despite the increased communication capacity, MPTCP is well-known to require a relatively large amount of end-system buffer memory space compared to the legacy TCP [6]. Especially when the two paths show diverse quality [7], e.g., in RTT or throughput, packets from a good quality path may arrive much faster than the ones from the poor quality path, resulting in out-of-order packets at the receiver. This is called the *buffer blocking problem*, which causes a large amount of buffering at both sender and receiver. This has shown to be a problem even for mobile phones [7], and it becomes exceedingly problematic for typically lower-memory IoT devices.

The MPTCP uses the standard BSD socket interface that hides the multipath transport from the application layer. Therefore, the application layer is generally unaware of MPTCP, thus transparent to the multipath transmissions. In this paper, we observe that this transparency is the key reason for the buffer blocking problem, and we opt to create multiple socket instances for the multipath transmission, instead of creating a single socket for multiple paths, so that the application can be aware of multipath transmissions. We use this idea to propose an application-level distributor that transmits packets to each socket at each endpoint of the data, i.e., starting point and end point of the data. This scheme cleverly avoids the buffer blocking problem, while still maintaining the benefits of multi-homing.

The main contribution of our paper is three-fold. First, we propose a low-memory multipath transfer scheme for IoT devices that exploits application layer multipath scheduling, so that the IoT device can achieve the benefits of a multipath while avoiding buffer blocking due to out-of-order packets. Second, our scheme utilizes the original MPTCP, and as a result, most of the modification is done at the application level. Thus, our proposal can easily be deployed to legacy systems. Finally, we provide experimental results using the Linux-based MPTCP test-bed, and show that our proposed scheme achieves equivalent performance to the legacy MPTCP while requiring only half of the memory space.

The rest of the paper is organized as follows. We explain the buffer blocking problem in Section 2. We then present our main proposal called Memory-Efficient Multipath Transmission in Section 3. In Section 4, we show our experimental results and Section 5 briefly reviews some of key related work. Finally, Section 6 concludes this paper.

## 2. Problem Statement

In MPTCP, the packets from the same connection traverse through multiple paths to reach the receiver. Each packet may arrive at the receiver not in the same order as they were originally sent, and as a result, out-of-order packets frequently occur. Therefore, MPTCP maintains two types of sequence numbers, namely *subflow sequence* and *data sequence*, to provide reliability and global ordering. Each subflow maintains their own subflow sequence to conduct both loss detection and retransmissions on each subflow just as a single TCP does. The MPTCP scheduler stripes the data packets from the output queue, and places them on each subflow according to the scheduling policy, and then, the MPTCP receiver uses the data sequence number to globally reassemble the data stream at the receiver buffer [6].

**Figure 1:**
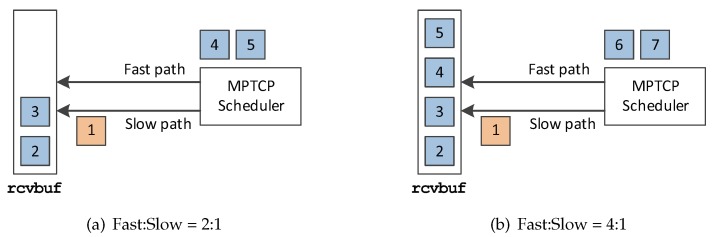
An example of the buffer blocking problem [Fast:Slow] shows the the relative speeds of the two paths. The left subfigure shows that the fast path is two times faster, while the right subfigure shows that the fast path is four times fast.

Since each subflow path may have diverse RTTs, the packets may be received out-of-order in terms of the data sequence number at the MPTCP receiver. This is especially challenging when the two paths have diverse quality. Let us give a simplified example to describe this phenomenon. If the speed (which is inversely proportional to RTT) ratio of the two paths is 2:1, there would be usually two out-of-order packets in the receive buffer (i.e., rcvbuf) as shown in Figure 1a, until the packet that fills the hole arrives from the slow path. One outcome of this out-of-order problem is the exhaustion of the receiver’s receive window, thus temporally stalling the transmission. In Figure 1b, the slow path is so *slow* that the fast path cannot transmit more data packets as the rcvbuf becomes full, which results in throughput degradation. We will show that this phenomenon occurs more frequently as the receive buffer size decreases in the Section 4.

To solve this problem, opportunistic retransmission & penalization allows to resend the unacknowledged data from the slow path to the fast path, and penalizes the slow path each time a stall happens [6]. These mechanisms are somewhat effective, however, but does not fully solve the problem of low-memory IoT devices as will be shown in Section 4.

## 3. Memory-Efficient Multipath Transmission

**Figure 2:**
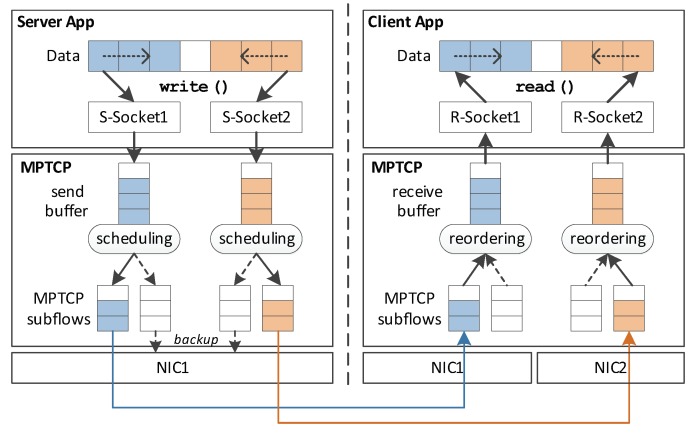
The proposed architecture of MPTCP-IoT. The left part of the architecture shows the operation of the MPTCP-IoT Server, while the right part shows that of the MPTCP-IoT Client. Each application creates multiple socket instances for multipath transmission, and only the main path of each socket is utilized for transmission (reception) at the Server (Client).

To address the problem described in the previous section, we design a low-memory multipath transmission scheme that requires small receive (and also send) buffers, while maintaining the benefits of MPTCP. The key design principle of our approach is to maintain multiple socket instances for the MPTCP connection, rather than keeping just one socket as the original MPTCP does. This reveals the multipath transmission to the application, thus can be leveraged to avoid out-of-order packets at the receiver. As shown in Figure 2, both the server and client maintains two sockets for the multipath connection.

For each socket instance, it utilizes only a single dedicated subflow (the main network path), rather than transmitting via all the available paths as the legacy MPTCP does. The other MPTCP subflow(s) would be used only as a backup mode. By doing so, we first avoid the out-of-order delivery, and second, keep the seamless handover feature as the backup subflow becomes active immediately when the dedicated subflow fails. Since our design allows each socket instance to communicate over its dedicated subflow, multiple socket instances are created to fully utilize multiple network paths concurrently. For this reason, we introduce an application-level data distributor among the socket instances.

Figure 2 depicts the overall architecture that shows this principle. We mainly consider a general scenario where a client has two network interfaces, e.g., 3G/4G and Wi-Fi; thus, two network paths are available between the client and server. According to our design principle, two socket pairs would be established, which are (S-Socket1, R-Socket1) and (S-Socket2, R-Socket2), followed by creating two dedicated subflows, each of which belongs to each socket-pair.

Next, we explain the operation of the proposed scheme in detail in the following two subsections, and then we present some discussions on the next subsection.

### 3.1. Server-Side Operation

The server-side operation is described in Algorithm 1. After the socket pairs are established, the server starts sending data using the data distributor. It divides the whole data into several small-sized chunks and sends each of them via either S-Socket1 or S-Socket2 (i.e., s1.socket and s2.socket in Algorithm 1). At this point, to avoid chunk-level data reordering, we perform write() from both end sides of the data; that is, processing the data chunks from the starting point in the forward direction for S-Socket1 and from the ending point in the reverse direction for S-Socket2, as shown in Figure 2 (lines 4–16 in Algorithm 1).
**Algorithm 1** Sender-side operations.*s_fd*: file descriptor of the sending file.*n*: total number of data chunks (file_size / chunk_size, n≥2).1: s1.index = 0;2: s2.index = n - 1;3: 4: *In S1-thread:*5: **repeat**6:  pread(s_fd, s1.buffer, chunk_size, s1.index); *// Read s1.index of chunk from fd*7:  write(s1.socket, s1.buffer, chunk_size); *// Send s1.index of chunk over s1.socket*8:  s1.index++;9: **until** s1.index ≥ s2.index10: 11: *In S2-thread:*12: **repeat**13:  pread(s_fd, s2.buffer, chunk_size, s2.index); *// Read s2.index of chunk from fd*14:  write(s2.socket, s2.buffer, chunk_size); *// Send s2.index of chunk over s2.socket*15:  s2.index–;16: **until** s1.index ≥ s2.index17: 18: *In MPTCP packet scheduler:*19: **if** current_socket == s1.socket **then**20:  **if** s1.subflow1 is active **then**21:   send_data(s1.subflow1, data);22:  **else if** s1.subflow2 is active **then**23:   send_data(s1.subflow2, data); *// s1.subflow2: backup-subflow of s1.socket*24:  **end if**25: **else if** current_socket == s2.socket **then**26:  **if** s2.subflow2 is active **then**27:   send_data(s2.subflow2, data);28:  **else if** s2.subflow1 is active **then**29:    send_data(s2.subflow1, data); *// s2.subflow1: backup-subflow of s2.socket*30:  **end if**31: **end if**

Next, at the MPTCP layer, each socket-level connection opens two MPTCP subflows; dedicated and backup. The dedicated subflows are chosen so that their network paths are different from each other. Then the packet scheduler *schedules* data packets only to the dedicated subflow unless it is unreachable or disconnected (lines 18–31 in Algorithm 1).

For example, S-Socket1 will use Wi-Fi as the main subflow and LTE as the backup, while S-Socket2 will use LTE as the main subflow and Wi-Fi as the backup. Since the application-level distributor will utilize both sockets, both paths will be fully utilized just as in full-mode MPTCP.

### 3.2. Client-Side Operation

Now, the receiver-side operation is described in Algorithm 2. When the data packets arrive at the subflow-level receive buffer of the client, they would go up to the MPTCP-level receive buffer immediately, without global sequence reordering. As a result, our design rarely uses the out-of-order buffer at the client-side and consumes relatively less memory compared to the conventional MPTCP.

Finally, the socket instances of the client application perform read() for the received data and similarly to the server-side operation, fill up the file from the starting point for R-Socket1 and from the ending point for R-Socket2 (i.e., r1.socket and r2.socket in Algorithm 2) through pwrite() (lines 4–16 in Algorithm 2).
**Algorithm 2** Receiver-side operations.*r_fd*: file descriptor of the receiving file.*n*: total number of data chunks (file_size / chunk_size, n≥2).1: r1.index = 0;2: r2.index = n - 1;3: 4: *In R1-thread:*5: **repeat**6:  read(r1.socket, r1.buffer, chunk_size); *// Receive r1.index of chunk from r1.socket*7:  pwrite(r_fd, r1.buffer, chunk_size, r1.index); *// Write r1.index of chunk to fd*8:  r1.index++;9: **until** r1.index ≥ r2.index10: 11: *In R2-thread:*12: **repeat**13:  read(r2.socket, r2.buffer, chunk_size); *// Receive r2.index of chunk from r2.socket*14:  pwrite(r_fd, r2.buffer, chunk_size, r2.index); *// Write r2.index of chunk to fd*15:  r2.index–;16: **until** r1.index ≥ r2.index

## 4. Evaluation

### 4.1. Discussions

We note that the client application is supposed to receive some information such as file and chunk sizes from the server before data transmission. This is feasible in many cases. For example, when using Web applications, the client can easily obtain the file size using the HTTP GET message [8]. Another example is when using HTTP based multimedia streaming, e.g., MPEG DASH [9], the chunk sizes can be obtained through the manifest file.

Although we have limited the number of interfaces to two in our examples, this can be fairly easily extended to more than two interfaces by dividing the file into more than two chunks. A similar approach was used in MP-H2 [8].

Note that our scheme used MPTCP sockets instead of TCP sockets, although only one interface is utilized at the same time for each socket. Therefore, one can argue that we should use the TCP socket instances instead of using MPTCP. However, in this paper, we focus on MPTCP as a transport protocol because of the following reasons: First, MPTCP supports user-mobility; the MPTCP sessions are not disconnected unless network connectivity is completely unavailable (when multiple network interfaces are available). Therefore a smart-watch user, for example, might be able to automatically *reconnect* to the Wi-Fi network without loss of service as long as the other connection (e.g., cellular networks) is maintained. In contrast, TCP was not designed for mobile users, thus the application has the responsibility for detecting available networks and reconnecting over the networks in the same scenario. Second, the standard TCP socket is not allowed to select the network interface; instead, TCP is always forced to use the default network interface according to the routing table, while MPTCP can utilize all the available interfaces at the same time without modifying the socket layer.

To evaluate our approach, we implement the proposed scheme in the Ubuntu 14.04 machines running MPTCP Linux kernel (version 0.90) [10]. Our implementation includes (i) the application-level data distributor and (ii) the MPTCP-level packet scheduler that creates dedicated subflows, and then we make a performance comparison with the conventional MPTCP over Wi-Fi and 3G networks, varying the socket buffer size.

### 4.2. Experimental Setup

**Figure 3:**
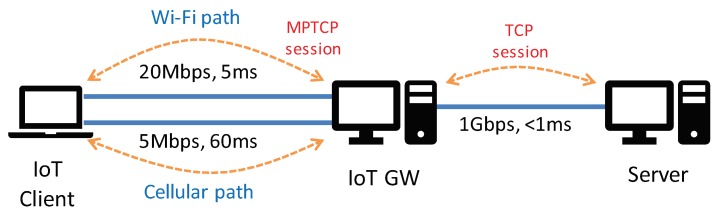
Testbed setup: The IoT client is connected to the IoT gateway (GW) via two emulated paths, i.e., Wi-Fi path and Cellular path, by MPTCP. The IoT GW is connected to the server via a TCP session.

First, we build a simple multipath topology that consists of three machines representing IoT client, IoT GW, and a server as shown in Figure 3. We have setup the testbed so that the server does not employ the MPTCP stack, but rather only runs the legacy TCP stack. To implement multipath, we deploy an IoT GW between the client and the server that establishes two separate sessions towards both directions: one for the MPTCP connection with the IoT client and the other for the TCP connection with the server. This is close to a realistic scenario typically deployed by telecommunication industries [4]. In this manner, the multi-homed IoT client can communicate with any servers regardless of whether the server is capable of MPTCP or not, while always benefiting from the multi-path connection [3]. To this end, we install Dante [11] in the IoT GW, a SOCKS server that relays packets between the two sessions, i.e., when the IoT client sends requests or data to the server, the packets are transmitted to the IoT GW first through the MPTCP connection, and then the SOCKS server forwards the incoming packets to the server via the TCP session, and vice versa.

For the wireless links between the IoT client and GW, we emulate Wi-Fi and 3G links with Dummynet [12]; we set 20 Mbps of bandwidth and 5 ms of one-way delay for the Wi-Fi link and 5 Mbps and 60 ms for the cellular (3G) link. For congestion control, we use CUBIC [13] for TCP connections and Linked Increases Algorithm (LIA) [14] for MPTCP connections. We also set to use the LowRTT-first packet scheduling algorithm for the conventional MPTCP scheme. For the TCP buffer size, we change the maximum receive/send buffer memory specified in tcp_rmem and tcp_wmem through the Linux proc filesystem, while enabling the TCP auto-tuning mechanism [15]. We note that the TCP connections between the IoT GW and the server should never be the bottleneck in all experiments as we provide enough buffer memory and network bandwidth to the TCP connections, in order to focus on the MPTCP performance over the wireless links. Finally, we use the best-effort type of traffic for all connections.

### 4.3. Throughput Measurement with Varying TCP Buffer Sizes

**Figure 4:**
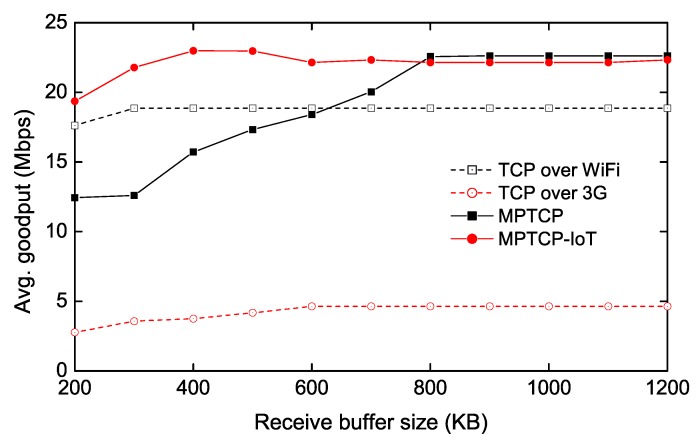
Throughput measurement as a function of receive-buffer size of IoT GW.

**Figure 5:**
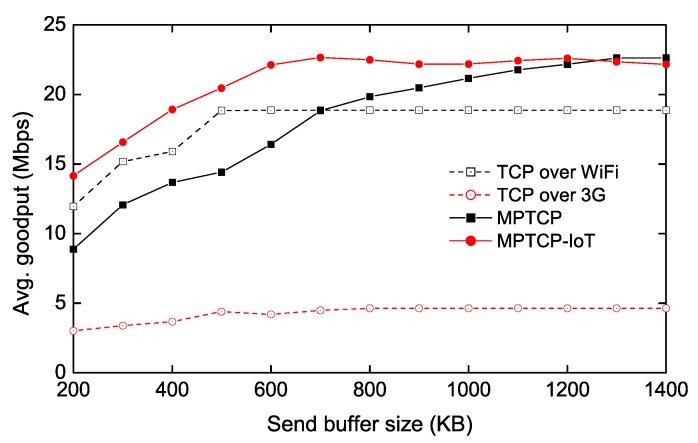
Throughput measurement as a function of send-buffer size of IoT device.

To evaluate how the proposed scheme performs under different TCP buffer sizes, we measure the average goodput as a function of the receive (send) buffer size while transmitting 10 MB data from the IoT client to the server (via the IoT GW), as shown in Figure 4 and Figure 5. Note that in our evaluations, we only present the performance of the upstream traffic (from client to server), since the downstream traffic (from server to client) shows very similar performance. Furthermore, IoT devices tend to use more upload traffic, since they generally produce more data to upload to the server. In Figure 4, we vary the receive buffer size of the IoT GW while setting the send buffer size of the IoT client to a sufficiently large number, so that the MPTCP receiver-side buffer becomes the bottleneck. We also measure the TCP throughput over each of Wi-Fi and 3G links as a guideline. We observe that MPTCP does not fully utilize both links until the receive buffer size reaches 800 KB. The main reason is that the congestion window size (cwnd) of the 3G path is kept small due to the penalization mechanism of MPTCP, caused by the buffer-blocking problem as described in Section 2. Furthermore, we also see that when the buffer size is less than 600 KB, MPTCP performs even worse than Wi-Fi-only TCP. Our analysis reveals that frequent buffer-blocking also leads to under-utilization on a fast path (i.e., Wi-Fi) because MPTCP has to stop transmitting on the fast path for a while to perform the opportunistic retransmission. On the other hand, the proposed scheme (denoted as MPTCP-IoT) requires much smaller size of the receive buffer to achieve the full aggregated throughput (i.e., TCP over Wi-Fi + TCP over 3G), since the packets received from the fast path do not need to wait for the slow packets at the out-of-order (receive) buffer. Therefore, we confirm that our approach is suitable for memory-limited systems when MPTCP is used.

We note that multipath transfers also require a higher send buffer size, as the sender needs to hold the transmitted packets in the send buffer until the corresponding acknowledgment packets are returned. To confirm this, similar to Figure 4, we vary the send buffer size of the IoT client while fixing the receive buffer size of the IoT GW to a large number in Figure 5. The result reveals that varying the send buffer affects the throughput more than the receive buffer for both TCP and MPTCP, requiring >1200 KB send buffer memory for the conventional MPTCP to achieve the best throughput. MPTCP-IoT also requires more memory to achieve its best throughput compared to the Figure 4 case, but this is because TCP over Wi-Fi and 3G throughput are degraded with a lower send buffer size (∼500 KB)—it achieves the full aggregated throughput with 600 KB send buffer that TCP over Wi-Fi (and 3G) shows the best throughput with.

### 4.4. Buffer Requirement with Varying RTTs

**Figure 6:**
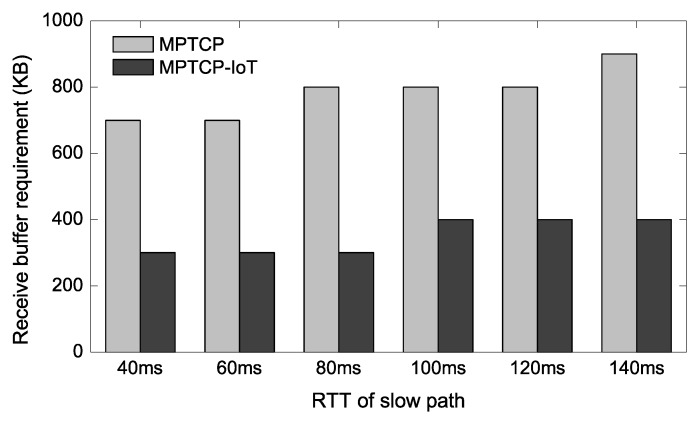
Receive-buffer requirement of IoT GW.

**Figure 7:**
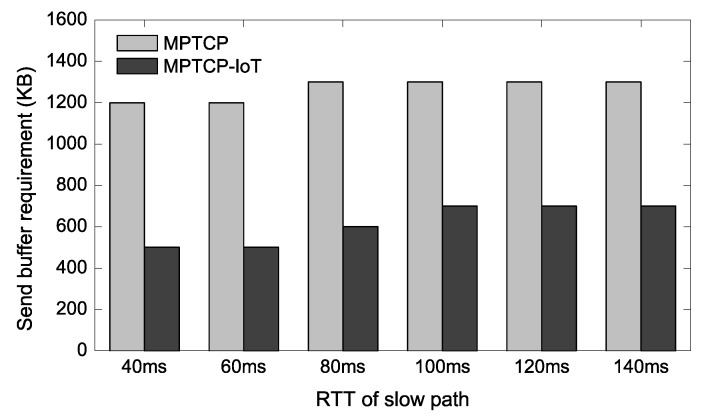
Send-buffer requirement of IoT device.

Now we vary the delay of the 3G link to confirm that the proposed scheme can reduce the buffer requirement generally when one of the available paths has higher latency. The other parameters remain unchanged from the experimental setup in Section 4.2. In Figure 6 and Figure 7, we show the buffer requirement for achieving the full aggregated throughput when either receive buffer of the IoT GW or send buffer of the IoT device is the bottleneck. Here, *buffer requirement* represents the minimum memory size required in order to achieve the same throughput as the case when a sufficiently large buffer memory is provided. In other words, the lesser the buffer requirement the better.

In Figure 6, we observe that the proposed scheme achieves 2.0–2.7× reductions over the conventional MPTCP in terms of the receive buffer requirement. Figure 7 shows a similar trend achieving about 1.9–2.4× send buffer memory reductions. This result is also consistent with Figure 5 as the send buffer bottleneck case requires up to 1.7× more memory compared to the receive buffer bottleneck case.

### 4.5. Throughput Measurement with Real Wi-Fi and 3G Networks

Finally, to demonstrate that the proposed scheme works well in real-world environments, we conduct a similar experiment using commercial Wi-Fi and 3G networks between the IoT client and GW.

We use an MPTCP server running in our university campus as the IoT GW. The IoT client is then connected to this server via a Wi-Fi network and a commercial 3G network. For this configuration, we use a Linux IoT client with Intel Core 2 Duo 2.4 GHz processor, 3 MB L2 cache, 2 GB DRAM, and 802.11 ac USB wireless network adaptor. We also add a smartphone to the IoT client using a wired cable, so that the client can be attached to a commercial 3G network via the tethering service.

**Figure 8:**
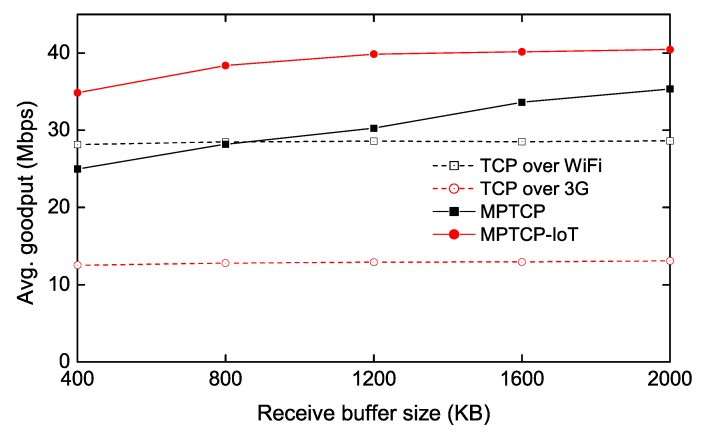
Throughput measurement on a real 3G/Wi-Fi topology.

The experimental result is shown in Figure 8. The average bandwidth is about 30 Mbps and 10 Mbps for Wi-Fi and 3G links, respectively. Figure 8 shows that the trend of the real measurement is very similar with that of the testbed measurement (Figure 4); the proposed scheme works well with a smaller size of the receive buffer while the conventional MPTCP requires higher receive buffer memory.

The final note is that, in our real-world experiments, we only measure the downlink throughput, as the uplink bandwidth of our 3G network is limited by the service provider’s capacity. Since changing the system parameters of the server (e.g., send buffer size) may affect the throughput of other TCP connections in the server, we vary only the receive buffer size at the IoT client in this experiment.

## 5. Related Work

This section briefly discusses some of the key related work including packet scheduling schemes and congestion control algorithms for multipath transfers.

*MPTCP Linux packet schedulers:* In [16], the authors compare the performance of the two basic schedulers implemented in the MPTCP Linux kernel, which are the Round-Robin (RR) and the Lowest-RTT-First (LowRTT). The main finding is that the LowRTT generally performs better than the RR scheduler in terms of the application delay-jitter. The BLEST [17] scheduler targets to reduce the sender-side blocking—i.e., the BLEST sender waits for the fast subflow to become available, so that the slow path’s packet does not block the connection-level send window.

*Advanced packet scheduling schemes:* Based on the LowRTT policy described above, several papers were proposed to modify the MPTCP scheduler. One proposal was to perform out-of-order transmissions at the sender-side for in-order arrivals [18]. It may require a small size of receive buffer even when the paths have diverse RTTs. However, it still entails a relatively large size of send buffer to keep the out-of-order packets in the send queue. ECF [19] computes the time to send the remaining traffic to each path, and utilizes the path that is expected to give the minimal flow completion time. These approaches somewhat reduces the reordering problem, but fails to alleviate them, while as our approach completely solves the reordering problem at the transport layer. MP-H2 [8] is similar to our proposal in a sense that the scheduling is done at the application layer. However, MP-H2 uses a receiver-based approach where the client makes all the scheduling decisions based on HTTP/2. MP-H2 completely disregards MPTCP, and heavily relies on the new features of HTTP/2, which is not yet deployed in many servers. Meanwhile, in our approach, we utilize the original MPTCP, and it does not rely on a specific application layer protocol, and furthermore, the intelligence remains at the server.

*MPTCP congestion control algorithms:* The initial approach for MPTCP congestion control was to simply adopt Reno’s algorithm on each subflow. However, this can hurt the TCP bandwidth as it is equivalent to creating multiple TCP flows per connection. To avoid such unfairness, one important design goal of MPTCP congestion control is to keep the friendliness towards TCP, which is achieved by moving as much traffic as possible off its most congested paths [14]. LIA (Linked-Increases Algorithm) is the stable algorithm that satisfies this design goal well and becomes the current default algorithm for MPTCP in the Linux implementation [14]. OLIA (Opportunistic Linked- Increases Algorithm), however, claims that there are some corner cases where LIA harms TCP bandwidth, thus is not pareto-optimal, and improves the performance addressing the problem [20]. The recent proposal, Balia (Balanced Linked Adaptation), shows that there is an inevitable tradeoff between friendliness to TCP and responsiveness to network dynamics, and strikes a good balance, mathematically proving that there is a unique and stable equilibrium point between the metric [21].

*Energy Efficiency of MPTCP:* Although MPTCP renders enhanced throughput to the client, it may still use more energy consumption. There has been some work that considers energy in MPTCP [22,23]. eMPTCP [22] uses power-aware subflow management and delayed subflow establishment to reduce the energy consumption of MPTCP. The work in [23] studies the energy performances of MPTCP under hard delay-constraints via numerical simulations by using the Settable-Complexity Bandwidth Manager (SCBM).

*Lightweight messaging protocols for IoT devices:* MQTT (Message Queuing Telemetry Transport) [24] is a publish/subscribe messaging protocol for machine to machine (M2M) communications. With MQTT, all clients (e.g., sensors) generally make a long-lived outgoing TCP connection to a server, which is called *broker*. Therefore, the nodes exchange their messages in an asynchronous manner; for example, clients can publish the messages even when the receiver node is not active, and similarly, the receivers can receive the messages from the broker when they become active. This allows the nodes to remain in the sleep-mode state even if there is a message to receive. On the other hand, CoAP (Constrained Application Protocol) [25] is a one-to-one protocol between clients and servers, but similar to MQTT, it supports the “observe” mechanism that enables the nodes to observe other nodes and queries some nodes for specific information when they awaken from the sleep-mode. Compared to these protocols, the main strength of the proposed scheme is supporting user-mobility with less memory requirement, which is helpful for many sensor devices (e.g., health-care devices attached to human bodies). In the energy perspective, we believe that our approach can be used with the above IoT protocols together. For example, IoT devices would be able to save energy by using MQTT/CoAP’s sleep-mode, while connecting to the other servers via our approach.

## 6. Conclusions and Future Work

Since the IoT devices are limited in computation power and memory, they generally connect to the cloud that provides a plethora of resources. To enhance the communication, IoT devices may use multihoming via MPTCP, but this causes extra buffer overhead to the IoT system. In this paper, we proposed a memory-efficient multipath transmission scheme for multi-homed low-memory IoT devices. Our scheme employs a application-level distributor, that cleverly avoids the buffer-blocking problem by revealing the multipath transmission to the application. Our Linux testbed and real-world experimental results show that the proposed scheme halves the required memory needed for multi-path transfer.

In this paper, we have shown that our proposal enhances the original MPTCP to cope with the low-memory IoT devices. However, our work still has several limitations and leave these issues as future work as follows:

First, our work can be improved by studying the impact of MPTCP congestion control algorithms. We have not modified any congestion control schemes used at the MPTCP layer [14,20,21] in our work. We leave the impact of these congestion control to our proposal as future work.

Second, reducing the energy consumption for IoT devices is a significant issue [23]. Our proposal reduces the transmission time of the IoT communication interfaces, thus increasing the sleep time of IoT devices to save energy. In our future work, we intend to present the numerical and experimental results of the energy consumption of the IoT devices to validate this argument.

Finally, we plan to conduct an in-depth performance comparison with MQTT [24] to better understand the energy consumption of the proposed scheme. In particular, we believe that our scheme and MQTT can complement each other. Therefore, we will try to figure out how much our scheme can save energy by co-working with MQTT.
